# Neuro-ophthalmic presentation of leptomeningeal metastasis of thymoma: a case report

**DOI:** 10.3389/fneur.2026.1788152

**Published:** 2026-02-25

**Authors:** Muhammad Hammad Khan, Syeda Fatima Abid, Dina Abdelsalam, Safa Ibrahim, Andrew G. Lee

**Affiliations:** 1 Department of Ophthalmology, King Edward Medical University, Lahore, Pakistan; 2 Department of Ophthalmology, Blanton Eye Institute, Houston Methodist Hospital, Houston, TX, United States; 3 Department of Ophthalmology, Cullen Eye Institute, Baylor College of Medicine, Houston, TX, United States; 4 Department of Ophthalmology, Neurology, and Neurosurgery, Weill Cornell Medicine, New York, NY, United States; 5 Department of Ophthalmology, University of Texas MD Anderson Cancer Center, Houston, TX, United States; 6 Texas A&M College of Medicine, Bryan, TX, United States; 7 Department of Ophthalmology, The University of Iowa Hospitals and Clinics, Iowa City, IA, United States

**Keywords:** cranial nerve palsy, leptomeningeal disease, metastatic thymoma, thymic carcinoma, thymoma

## Abstract

**Introduction:**

Leptomeningeal disease (LMD) of the brain and spinal cord can present with visual loss or diplopia. Although LMD can occur in many forms of neoplasia, thymoma-related LMD is exceedingly rare.

**Patient presentation:**

A 53-year-old Hispanic male with a history of chest pain, weight loss, and night sweats was diagnosed with stage 4 thymoma with lung and pleural metastasis. He received chemotherapy for metastatic thymoma. Few months later, patient presented with severe right-sided facial pain and lip numbness, ptosis and double vision.

**Primary diagnosis:**

The patient was diagnosed with multiple cranial and spinal nerve involvement due to thymomatous LMD, confirmed on magnetic resonance imaging and lumbar puncture.

**Conclusion and importance:**

LMD is a rare presentation of a malignant thymoma. Current guidelines for thymoma management emphasize the importance of staging imaging to rule out distant metastasis. Our case highlights the importance of a head-to-mid-thigh positron emission tomography (PET) scan in patients with known metastatic thymomas, with multiple PET scans, if possible, at regular intervals, owing to the aggressive nature of metastatic thymomas. Clinicians should be aware of the neoplastic (e.g., metastatic disease and LMD) and paraneoplastic (e.g., thymoma-related myasthenia gravis) neuro-ophthalmic presentations of thymoma.

## Introduction

Thymoma is an uncommon neoplasm with an incidence of 0.13–0.19 cases per 100,000 persons at risk (1, 2). Thymomas arise from the thymic epithelial cells located in the anterior mediastinum and can be benign or malignant. There is a strong association between thymoma and autoimmune disorders (e.g., myasthenia gravis, pure red cell aplasia, and Good syndrome) (3, 4), and may be due to dysfunction of T-cell activity in the context of abnormal maturation and differentiation of thymic cells in the neoplasm.

The incidence of malignant thymoma is rare, and cases with extra-thoracic spread have only been documented in a few case series and reports. Common sites of extra-thoracic metastasis include lymph nodes, liver, bones, and brain (5). Leptomeningeal disease (LMD) in thymic neoplasm represents a late-stage complication and has an extremely poor prognosis. A systematic review from 2017 mentions only 8 to 10 cases ever reported for brain and LMD (6). We report a case of thymomatous LMD presenting with diplopia and multiple cranial neuropathies.

## Clinical findings

A 53-year-old man presented with acute, binocular horizontal diplopia, right-sided headache, and facial numbness. Past medical history included a diagnosis of malignant thymoma, with extension to the lungs and pleura. Surgical history was non-contributory, as the patient’s thymoma had been deemed unresectable. The patient was taking the following medications: albuterol for asthma, which was well-controlled, and gabapentin for generalized pain and myalgias. Social history included smoking (3.2 pack years), although he had quit 6 years ago; a history of alcohol abuse, with 10 standard alcoholic drinks per week, but none at presentation. The patient developed constitutional symptoms and chest pain. Patient’s family history was unremarkable for thymic epithelial neoplasm.

Chest X-ray, chest computed tomography (CT), and magnetic resonance imaging (MRI) of the chest revealed total collapse of the left lower lobe, extensive mass effect upon the left upper lobe, and bulky lymphadenopathy of the anterior mediastinum consistent with thymoma. Full-body positron emission tomography (PET) scan confirmed hyperavidity in the mediastinal mass, suggestive of malignancy. However, at this stage, no suspicious brain or lymph node uptake in the head and neck region was noted on the PET scan.

Endobronchial ultrasound-guided biopsy showed lymphoepithelial cells consistent with a thymoma. Staging body imaging showed stage 4 thymoma with lung and pleural metastasis. The patient was started on cisplatin, doxorubicin, and cyclophosphamide for six cycles. Follow-up imaging of the lung and pleural metastasis showed a good response to the chemotherapy.

**Figure 1:**
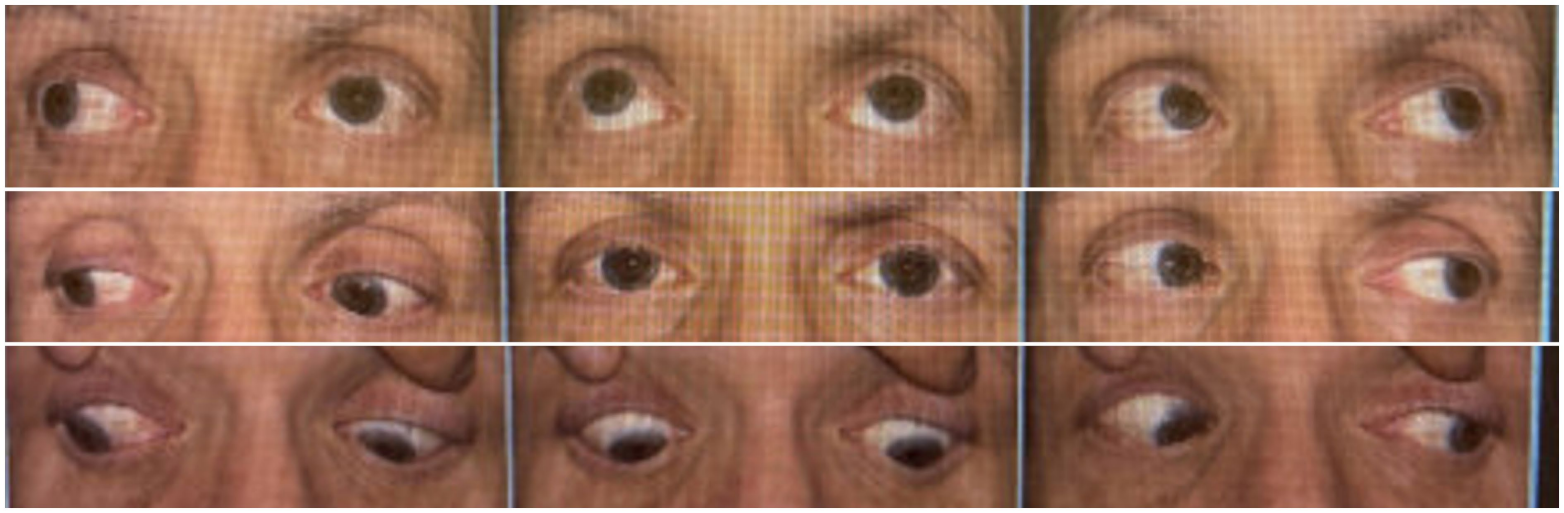
Patient’s Nine-gaze, showing marked extraocular movement restriction.

However, two months later, the patient presented to the emergency department with severe right-sided facial pain and lip numbness (cranial nerve V), and bilateral lower extremity discomfort and myalgias. This was followed 2 weeks later by ptosis and double vision. On examination, he was alert and oriented to person, place, time, and situation, and was able to follow complex commands. There was marked numbness in the V1 and V2 distribution of the trigeminal nerve on the right side, with decreased corneal sensitivity. The left eye (OS) showed limited adduction along with limited elevation and depression in abduction, suggestive of a cranial nerve III palsy OS. There was also a lack of torsion in downgaze consistent with concomitant cranial nerve IV involvement OS (Figure 1). Rest of the cranial nerve examination was unremarkable. Motor exam revealed equal and normal muscle bulk, tone and strength in lower as well as upper extremities. Deep tendon reflexes were intact throughout. Sensations were intact and symmetric in all extremities to light touch, pinprick, temperature, vibration, and proprioception.

**Figure 2:**
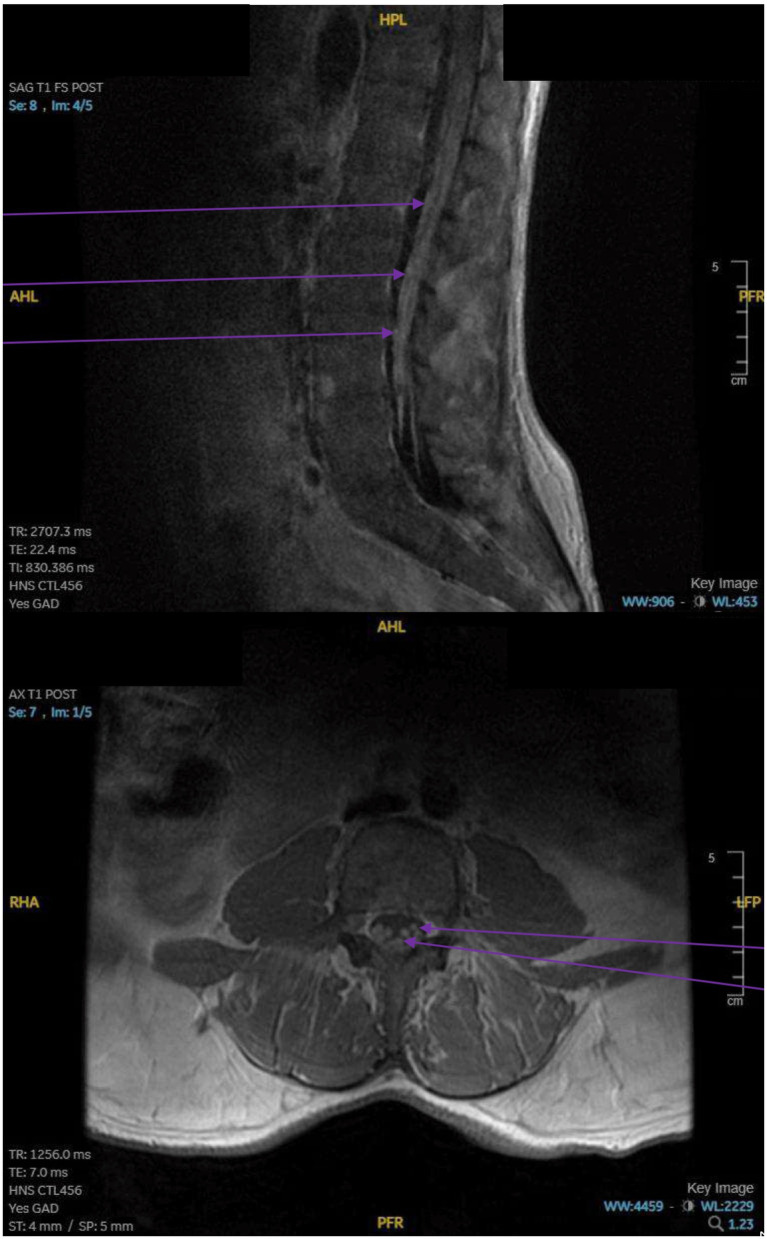
Saggital and Axial MRI of Lumbar Spine, showing distinct nodular metastasis (Purple Arrow).

**Figure 3:**
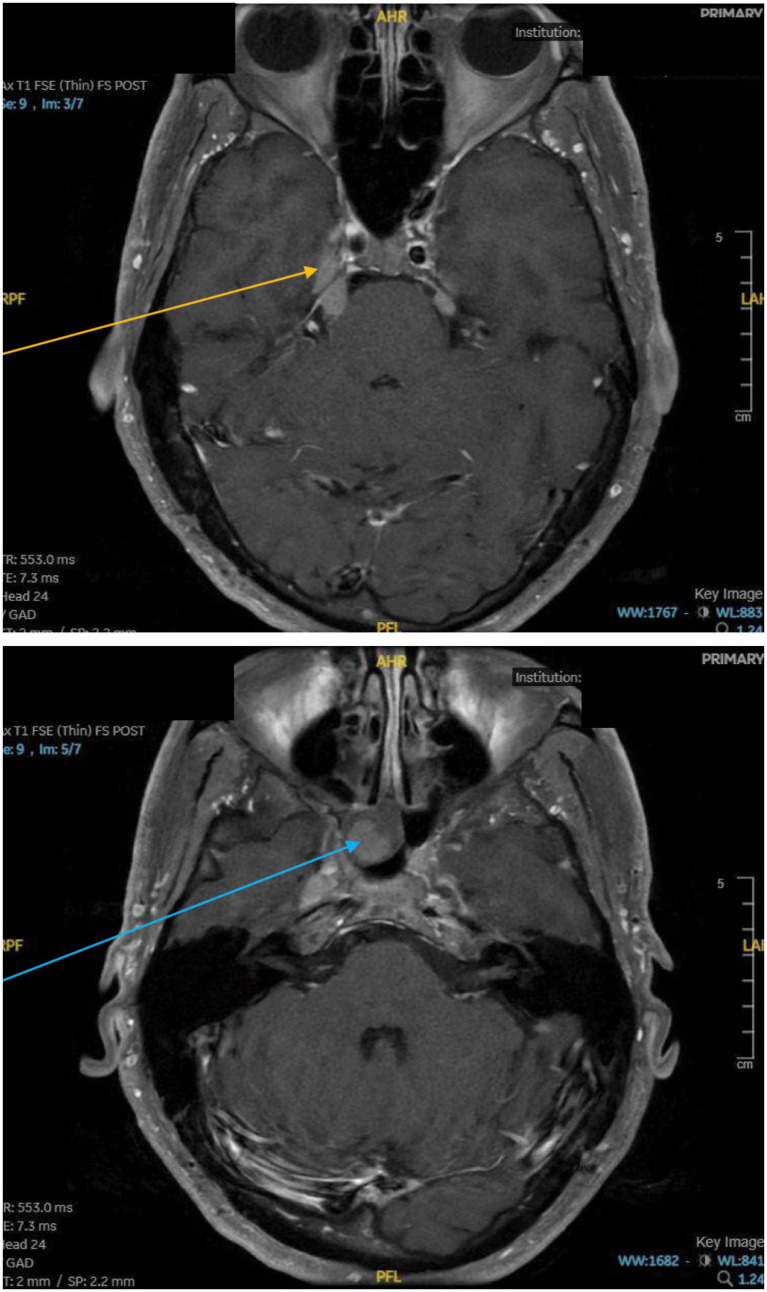
Axial MRI of Internal Auditory Canal showing enhancement within the right Meckel’s cave (Orange arrow), and Right Cranial Nerve V3 segment (Blue arrow).

**Figure 4:**
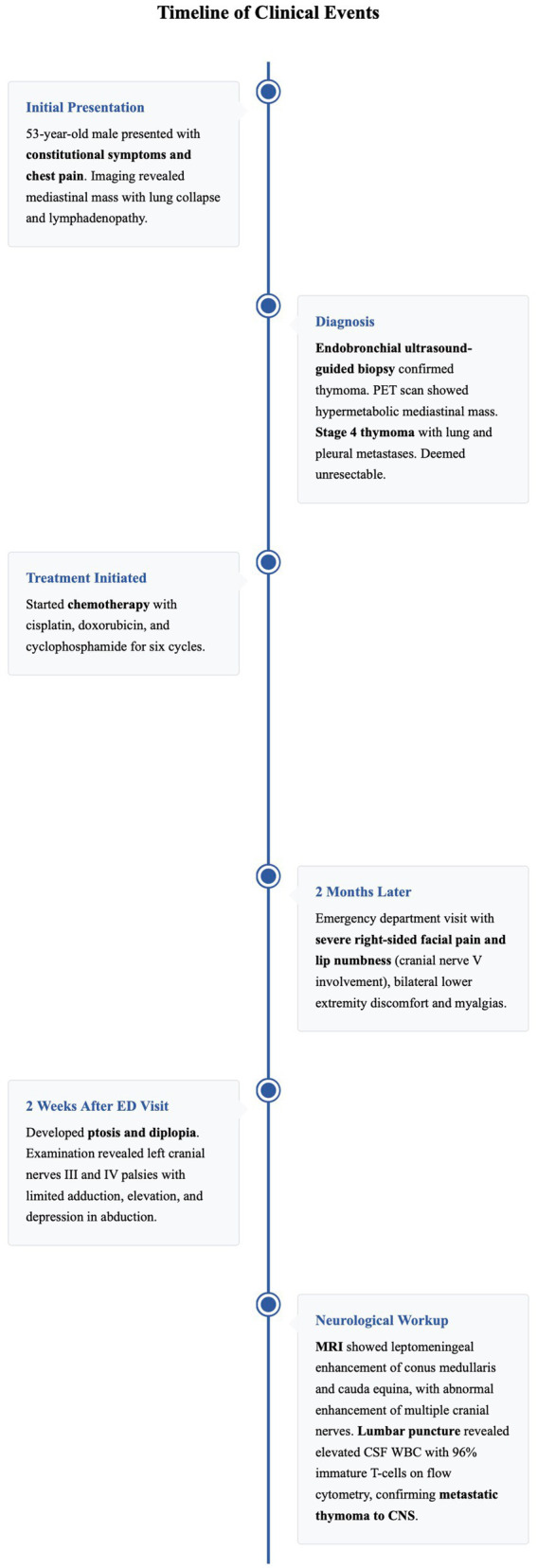
Timeline of clinical events.

Laboratory testing for myasthenia gravis was negative. The clinical picture prompted suspicion of intracranial spread that was responsible for the cranial nerve palsies. MR imaging of the head, neck, and spine revealed nodular leptomeningeal enhancement involving the conus medullaris and cauda equina in the spine (purple arrows in Figure 2). In addition, there was abnormal enhancement involving multiple cranial nerves in the posterior cranial fossa, the right Meckel’s cave (orange arrow), and the right cranial nerve V3 segment (blue arrow), as shown in Figure 3. The lower extremity pain was well accounted for by the leptomeningeal disease of the spinal cord. In addition, the patient’s presentation of right sided facial pain and numbness was adequately described by intracranial nodular metastases involving the trigeminal nerve. However, this mass lesion did not adequately account for the cranial nerve palsies seen on the left side. Thereafter, upon detailed review by the neuroradiology team, involvement of multiple cranial nerve palsies (cranial nerves III and IV on the left side) in the absence of a visible mass was deemed more likely due to leptomeningeal metastasis of thymoma. A lumbar puncture was ordered to confirm the diagnosis of leptomeningeal disease in this setting (see Figure 4).

Lumbar puncture revealed a markedly elevated cerebrospinal fluid protein of 593 mg/dL and albumin content measuring 398 mg/dL. The white blood cell count was notably high, at 2,294 cells/mm^3^ with differential count showing 43% lymphocytes and 57% monocytes. Flow cytometry showed these lymphocytes comprised of 96% immature T-cells, consistent with metastatic thymoma.

Thus, the patient was formally diagnosed with intracranial and spinal metastases of thymoma. Thereafter, because of the need to target specific neoplastic lesions in the brain and spinal cord, the patient underwent whole brain radiation and 10 fractions of 30 Gy radiation to the lumbar spine, which was overseen by the radiation oncology department.

The patient was understanding of the change in therapeutic regimen, from chemotherapy to radiotherapy, and understood that surgery could not be attempted in his case owing to the extensive metastases. His primary concerns were regaining normal vision and relieving the pain. It was explained to him that his presentation was exceedingly rare, and while radiotherapy could ideally help achieve these goals, the outcomes could vary widely.

## Discussion

LMD due to metastatic thymoma is exceedingly rare and based upon our review of the literature has only been described nine times previously (Table 1). However, there have been no prior reports of simultaneous involvement of the brain and spinal cord from thymoma metastasis. The World Health Organization (WHO) classifies thymomas into five types, designated as Type A, AB, B1, B2, and B3, based on cellular morphology and the presence of lymphocytic components (7). According to the National Cancer Collaborative Network (NCCN), diagnostic workup for thymic masses involves screening for paraneoplastic syndromes, chest imaging with CT/MRI and fluorodeoxyglucose PET scan from skull base to mid-thigh (8). Thymoma can produce diplopia from intracranial metastatic disease, LMD, or from thymoma associated myasthenia gravis.

**Table 1:** Previously reported cases of thymoma metastasis to leptomeninges and brain.

Study ID	Age of presentation and sex	WHO classification	Treatment of primary mass	Treatment of brain/LMD metastasis	Outcomes/survival
Belaid et al. (9)	54 years/female	—	Cyclophosphamide, doxorubicin, and cisplatin + 60 Gy radiotherapy to anterior mediastinum	Intrathecal cytarabine (refused)	Patient became extremely aggressive, and refused to take any medication
Gharwan et al. (6)	39 years/male	Subtype B3	Cisplatin, doxorubicin, cyclophosphamide, milciclib, sunitinib	—	—
Gharwan et al. (6)	31 years/female	Subtype B3	Neoadjuvant cisplatin, doxorubicin, cyclophosphamide, surgery, cisplatin/etoposide, RT	Radiotherapy	11 months after diagnosis of brain metastasis
Haryu et al. (10)	55 years/male	Subtype B3	Surgical resection and 40 Gy radiation + chemotherapy	Surgery	Improvement of hemiparesis
Vladislav et al. (11)	45 years/male	Subtype B1/2	—	—	—
Ohata et al. (12)	48 years/female	Subtype AB	Surgical resection	Surgery	Disease remission at 5 years follow-up
McLaughlin et al. (13)	22 years/female	Subtype B3	Surgical resection and 50 Gy radiation postoperatively	Surgery plus stereotactic radiosurgery	Disease remission at 32 months follow-up
Gamboa et al. (14)	78 years/female	Subtype A	Surgical resection	Whole brain radiotherapy + stereotactic surgery	Death at 17 months after surgery
Thompson et al. (15)	45 years/male	—	Ifosfamide 4 cycles + surgical resection due to poor response	Craniotomy and subtotal resection + 9 fractions of whole brain radiation	Mild condition improvement initially, followed by decline and death at the age of 46 years
Kanayama et al. (16)	80 years/male	Subtype B2	—	Surgery (trans-sphenoidal resection)	Death at 6 months after surgery
Adhikari et al. (17)	51 years/male	No subtype	—	Steroid therapy	Death at 8 months after surgery
Dewes et al. (18)	49 years/male	No subtype	Radiation	Surgery plus radiotherapy	Disease remission at 1 year follow-up

Our case report adds value to current literature on thymoma and its variable presentations by emphasizing the need for extensive imaging in patients at initial presentation. The more common paraneoplastic presentations of thymoma, such as myasthenia gravis, can mask the more malignant manifestations, such as intracranial and spinal metastases. Thus, a comprehensive metabolic, autoimmune, paraneoplastic, and radiographic image should be constructed for such a patient. In our case, the patient had not been seen by a physician in over a decade before presenting with severe chest pain, when he was diagnosed with pleural and pulmonary metastatic disease. Therefore, one of the limitations of our case report is the limited knowledge of the point in time when the patient developed leptomeningeal disease, which would be needed to construct a detailed timeline showing the relation of disease development to presentation.

Clinicians should be aware that thymoma can be benign or malignant and that malignant thymoma can metastasize. Multiple and bilateral cranial nerve presentations (including facial pain and ophthalmoplegia) should prompt consideration for LMD. The prognosis for LMD is generally poor and thymomatous LMD is exceedingly rare.

## Data Availability

The datasets presented in this article are not readily available because of ethical and privacy restrictions. Requests to access the datasets should be directed to the corresponding author.

## References

[1] Hsu C-H Chan JK Yin C-H Lee C-C Chern C-U Liao C-I. Trends in the incidence of thymoma, thymic carcinoma, and thymic neuroendocrine tumor in the United States. PLoS One. (2019) 14:e0227197. doi: 10.1371/journal.pone.0227197, 31891634 PMC6938371

[2] Robinson SP Akhondi H. "Thymoma" In: StatPearls. Treasure Island, FL: StatPearls Publishing (2025)

[3] Zhao J Bhatnagar V Ding L Atay SM David EA McFadden PM . A systematic review of paraneoplastic syndromes associated with thymoma: treatment modalities, recurrence, and outcomes in resected cases. J Thorac Cardiovasc Surg. (2020) 160:306–314.e14. doi: 10.1016/j.jtcvs.2019.11.052, 31982129

[4] Hashimoto S Hayasaka K Suzuki K Endoh M Yanagawa N Shiono S. Thymic small cell carcinoma associated with Lambert–Eaton myasthenic syndrome. Ann Thorac Surg. (2020) 109:e347–8. doi: 10.1016/j.athoracsur.2019.08.080, 31586614

[5] Engels EA. Epidemiology of thymoma and associated malignancies. J Thorac Oncol. (2010) 5:S260–5. doi: 10.1097/JTO.0b013e3181f1f62d, 20859116 PMC2951303

[6] Gharwan H Kim C Thomas A Berman A Kim SA Biassou N . Thymic epithelial tumors and metastasis to the brain: a case series and systematic review. Transl Lung Cancer Res. (2017) 6:588–99. doi: 10.21037/tlcr.2017.08.06, 29114474 PMC5653528

[7] Marx A Chan JKC Chalabreysse L Dacic S Detterbeck F French CA . The 2021 WHO classification of tumors of the thymus and mediastinum: what is new in thymic epithelial, germ cell, and mesenchymal tumors? J Thorac Oncol. (2022) 17:200–13. doi: 10.1016/j.jtho.2021.10.010, 34695605

[8] Riely GJ Wood DE Loo BW Aisner DL Akerley W Bauman JR . Thymomas and thymic carcinomas, version 2.2025, NCCN clinical practice guidelines in oncology. J Natl Compr Cancer Netw. (2025) 23:255–69. doi: 10.6004/jnccn.2025.0027, 40499586

[9] Belaid I Karrit S Fatma LB Ezzairi F Ammar N Chabchoub I . Exclusive meningeal relapse of a malignant thymoma after a complete response with neoadjuvant chemotherapy. Acta Neurol Belg. (2020) 120:409–11. doi: 10.1007/s13760-018-0904-1, 29468565

[10] Haryu S Saito A Inoue M Sannohe S Kurotaki H Kon H . Brain metastasis from invasive thymoma mimicking intracerebral hemorrhage: case report. Neurol Med Chir. (2014) 54:673–6. doi: 10.2176/nmc.cr2012-0430, 24305012 PMC4533487

[11] Vladislav T Jain RK Alvarez R Mehta RJ Gökmen-Polar Y Kesler KA . Extrathoracic metastases of thymic origin: a review of 35 cases. Mod Pathol. (2012) 25:370–7. doi: 10.1038/modpathol.2011.178, 22080058

[12] Ohata N Usami N Kawaguchi K Tateyama H Yokoi K. Type AB thymoma with brain metastasis: report of a case. Surg Today. (2011) 41:1436–8. doi: 10.1007/s00595-010-4442-6, 21922373

[13] McLaughlin SS Peckham SJ Enis JA Koebbe C Smith BD. Young woman with thymoma metastatic to the brain controlled with gross total resection and stereotactic radiosurgery, with a subsequent uncomplicated pregnancy. J Clin Oncol. (2011) 29:e30–3. doi: 10.1200/JCO.2010.31.0003, 20940185

[14] Gamboa EO Sawhney V Lanoy RS Haller NA Powell AT Hazra SV. Widespread metastases after resection of noninvasive thymoma. J Clin Oncol. (2008) 26:1752–5. doi: 10.1200/JCO.2007.14.5656, 18375905

[15] Thompson EM Sather MD Reyes CA Long DJ. Intracranial leptomeningeal metastasis from thymic carcinoma: case report and review. Surg Neurol. (2007) 68:233–8. doi: 10.1016/j.surneu.2006.08.079, 17537485

[16] Kanayama S Matsuno A Nagashima T Ishida Y. Symptomatic pituitary metastasis of malignant thymoma. J Clin Neurosci. (2005) 12:953–6. doi: 10.1016/j.jocn.2004.11.020, 16326276

[17] Adhikari MR Pereira P Ramesh M Bhat R. Malignant thymoma with cerebral metastases in association with pure red cell aplasia. J Assoc Physicians India. (1994) 42:245–6. 7860519

[18] Dewes W Chandler WF Gormanns R Ebhardt G. Brain metastasis of an invasive thymoma. Neurosurgery. (1987) 20:484. doi: 10.1227/00006123-198703000-00024, 3553984

