# Supplementary material for: Neuro-ophthalmic presentation of leptomeningeal metastasis of thymoma: a case report
Source: Front Neurol. 2026 Feb 25;17:1788152. doi: 10.3389/fneur.2026.1788152 (PMC12975580; doi:10.3389/fneur.2026.1788152)
Supplement: Supplementary file 1 [file Data_Sheet_1.pdf]

CARE Checklist of information to include when writing a case report

CARE case report guidelines

(cc) BY-NC-ND

Topic	Item	Checklist item description	Reported on Line
Title	1	The diagnosis or intervention of primary focus followed by the words "case report"	Title Page
Key Words	2	2 to 5 key words that identify diagnoses or interventions in this case report, including "case report"	Page 2: Keywords
Abstract (no references)	3a	Introduction: What is unique about this case and what does it add to the scientific literature?	Abstract: Introduction
	3b	Main symptoms and/or important clinical findings	Abstract: Patient presentation
	3c	The main diagnoses, therapeutic interventions, and outcomes	Abstract: primary diagnoses
	3d	Conclusion—What is the main "take-away" lesson(s) from this case?	Abstract: conclusion
Introduction	4	One or two paragraphs summarizing why this case is unique (**may include references**)	Introduction
Patient Information	5a	De-identified patient specific information.	Clinical findings
	5b	Primary concerns and symptoms of the patient.	Clinical findings
	5c	Medical, family, and psycho-social history including relevant genetic information	Clinical findings
	5d	Relevant past interventions with outcomes	Clinical findings
Clinical Findings	6	Describe significant physical examination (PE) and important clinical findings.	Clinical findings
Timeline	7	Historical and current information from this episode of care organized as a timeline	Provided as image
Diagnostic Assessment	8a	Diagnostic testing (such as PE, laboratory testing, imaging, surveys).	Clinical findings
	8b	Diagnostic challenges (such as access to testing, financial, or cultural)	-
	8c	Diagnosis (including other diagnoses considered)	Clinical findings
	8d	Prognosis (such as staging in oncology) where applicable	Clinical findings
Therapeutic Intervention	9a	Types of therapeutic intervention (such as pharmacologic, surgical, preventive, self-care)	Clinical findings
	9b	Administration of therapeutic intervention (such as dosage, strength, duration)	Clinical findings
	9c	Changes in therapeutic intervention (with rationale)	Clinical findings
Follow-up and Outcomes	10a	Clinician and patient-assessed outcomes (if available)	Clinical findings
	10b	Important follow-up diagnostic and other test results	Clinical findings
	10c	Intervention adherence and tolerability (How was this assessed?)	Clinical findings
	10d	Adverse and unanticipated events	-
Discussion	11a	A scientific discussion of the strengths AND limitations associated with this case report	Discussion
	11b	Discussion of the relevant medical literature **with references**.	Discussion; Table 1
	11c	The scientific rationale for any conclusions (including assessment of possible causes)	Discussion
	11d	The primary "take-away" lessons of this case report (without references) in a one paragraph conclusion	Discussion
Patient Perspective	12	The patient should share their perspective in one to two paragraphs on the treatment(s) they received	Clinical findings
Informed Consent	13	Did the patient give informed consent? Please provide if requested	Yes ☑ No ☐
